# Discovery and Characterization of Bacteriophage LuckyBarnes

**DOI:** 10.1128/MRA.00330-19

**Published:** 2019-06-20

**Authors:** Savannah L. Underwood, Amanda Foto, Amanda F. Ray, Alexander E. Nelms, Keyshawn L. Kennedy, Shelby G. Hartley, Logan M. Ryals, Chandan Gurung, William A. D’Angelo, Welkin H. Pope, Dmitri V. Mavrodi

**Affiliations:** a Department of Cell and Molecular Biology, School of Biological, Environmental, and Earth Sciences, The University of Southern Mississippi, Hattiesburg, Mississippi, USA; b Department of Biological Sciences, University of Pittsburgh, Pittsburgh, Pennsylvania, USA; Queens College

## Abstract

Here, we report the genome sequence of LuckyBarnes, a newly isolated singleton siphovirus that infects Brevibacterium iodinum ATCC 15728 and has a 50,774-bp genome with 67 predicted genes.

## ANNOUNCEMENT

*Actinobacteria* encompass saprophytes, commensals, and pathogens, as well as producers of anticancer, anthelmintic, and antifungal secondary metabolites (1). Bacteriophages play a crucial role in the evolution of *Actinobacteria* and provide insights into the genetics and physiology of this economically important group of bacteria. Despite the apparent importance of actinobacteriophages, our understanding of their biology is limited to viruses of *Mycobacterium*, *Gordonia*, and *Arthrobacter*, while the knowledge of phages that infect other members of *Actinobacteria* is lagging (2). We report here the characterization of a new bacteriophage, LuckyBarnes, which was isolated from soil collected in D’Iberville, MS, using enrichment with Brevibacterium iodinum ATCC 15728 in a peptone yeast calcium (PYCa) medium. Genome analysis identified LuckyBarnes as a singleton siphovirus and as one of the only two *Brevibacterium* phages with sequenced genomes in the Actinobacteriophage database (3).

**FIG 1:**
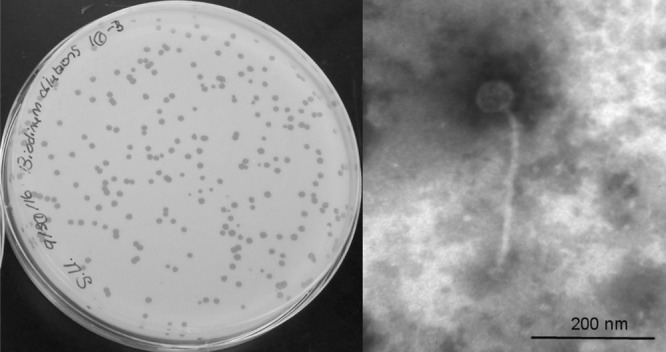
Plaque appearance (left) and virion morphology (right) of bacteriophage LuckyBarnes. For electron microscopy, high-titer lysate was applied on Formvar-coated grids, negatively stained with 1% phosphotungstic acid, and imaged with a Zeiss EM-109 transmission electron microscope (Carl Zeiss AG).

The bacteriophage was recovered by passing the enrichment culture through a 0.22-μm filter and incubating the filtrate with *B. iodinum* at room temperature for 48 h, which resulted in 1-mm clear plaques. Transmission electron microscopy of LuckyBarnes revealed a 50-nm capsid and a 250-nm-long flexible tail (Fig. 1). Phage DNA was isolated using the Wizard DNA cleanup kit (Promega) and used to prepare a library with a NEBNext Ultra II FS kit (New England BioLabs), which was then sequenced on an Illumina MiSeq instrument with MiSeq v3 chemistry. The sequence run generated 1.42 million 150-bp single-end reads that were assembled in Newbler v.2.9 (4), with default parameters, to give a 50,774-bp contig with 3,954-fold coverage and a G+C content of 61.9%. No defined genomic termini could be identified, and to preserve gene contiguity, the genome start point was selected 2.74 kb upstream of the predicted terminase gene.

The genome sequence was analyzed with GeneMark v.3.25 (5), GLIMMER (6), tRNAscan-SE v.2.0 (7), and ARAGORN v.1.2.38 (8), followed by manual annotation using DNA Master v.5.0.2 (http://cobamide2.bio.pitt.edu/computer.htm), Starterator (https://seaphages.org/software/), and HHpred (9). Phamerator (10) analysis of the 67 predicted protein-coding genes revealed that most of them (86%) lack homologs in other actinobacteriophages. Several structural genes were similar to their counterparts from the *Arthrobacter* phage TripleJ (https://phagesdb.org/phages/TripleJ/). The genes account for a 94.7% coding capacity of the genome and are arranged into two genome arms transcribed in divergent directions, which is similar to cluster A mycobacteriophages (11). The 29.6-kb left genome arm contains virion structure and assembly genes, followed by genes for a terminase, a portal protein, a tail terminator, a tape measure protein, a major tail subunit, minor tail proteins, and lytic enzymes represented by a class II holin and an *N*-acetylmuramoyl-l-alanine amidase. Genes within the 20.6-kb right genome arm encode several DNA metabolism enzymes, including a DNA polymerase, a primase, a DNA helicase, a single-stranded (ssDNA) binding protein, as well as a predicted dCMP-hydroxymethylase and two glycosyltransferases that may modify its DNA and improve resistance to degradation by host nucleases. The rest of the predicted genes (42%) encode conserved hypothetical proteins. Clear plaques and the absence of an integrase gene suggest that LuckyBarnes is a lytic phage.

### Data availability.

The genome of bacteriophage LuckyBarnes was deposited in DDBJ/ENA/GenBank under the accession number MF668275. The raw reads are available in the SRA under accession number SRR8782908.
